# AMPK-PINK1/Parkin Mediated Mitophagy Is Necessary for Alleviating Oxidative Stress-Induced Intestinal Epithelial Barrier Damage and Mitochondrial Energy Metabolism Dysfunction in IPEC-J2

**DOI:** 10.3390/antiox10122010

**Published:** 2021-12-18

**Authors:** Shuting Cao, Hao Xiao, Xin Li, Jiang Zhu, Jingchun Gao, Li Wang, Caihong Hu

**Affiliations:** 1 State Key Laboratory of Livestock and Poultry Breeding, Ministry of Agriculture Key Laboratory of Animal Nutrition and Feed Science in South China, Guangdong Key Laboratory of Animal Breeding and Nutrition, Maoming Branch, Guangdong Laboratory for Lingnan Modern Agriculture, Institute of Animal Science, Guangdong Academy of Agricultural Sciences, Guangzhou 510640, China; caoshuting@gdaas.cn (S.C.); xiaohao@gdaas.cn (H.X.); gaojingchun2021@126.com (J.G.); 2 Key Laboratory of Animal Feed and Nutrition of Zhejiang Province, Animal Science College, Zhejiang University, Hangzhou 310058, China; lixin_andy@163.com (X.L.); zhujiang0205@126.com (J.Z.)

**Keywords:** oxidative stress, intestinal barrier function, mitochondrial energy metabolism, mitophagy, AMPK-PINK1/Parkin

## Abstract

The imbalance of redox biology and oxidative stress leads to intestinal barrier injury and mitophagy. However, much uncertainty still exists about the role of mitophagy in oxidative stress and intestinal function. Here, we showed the effects of hydrogen peroxide (H_2_O_2_)-induced oxidative stress on intestinal epithelial cell oxidation balance, intestinal barrier function and mitochondrial energy metabolism and its underlying mechanism. In this study, we found that H_2_O_2_-induced oxidative stress activated adenosine monophosphate-activated protein kinase (AMPK) and enhanced mitophagy in intestinal porcine epithelial cells (IPEC-J2). While compound C (AMPK inhibitor) and mdivi-1 (mitophagy inhibitor) significantly reduced the activity of superoxide dismutase (SOD) and increased mitochondrial reactive oxygen species (ROS) levels in H_2_O_2_ treated cells. Moreover, compound C and mdivi-1 significantly reduced the trans-epithelium electrical resistant (TER) and increased the fluorescein isothiocyanate-dextran (FD4) flux in H_2_O_2_ treated IPEC-J2. Furthermore, compound C and mdivi-1 significantly reduced the activity of mitochondrial complex II. Seahorse XF96 data showed that compound C + mdivi-1+ H_2_O_2_ treatment significantly reduced maximum respiratory oxygen consumption and spare respiratory capacity. Additionally, compound C or mdivi-1 treatment reduced the formation of mitochondrial autophagosomes. These results unveiled that AMPK and PINK1/Parkin mediated mitophagy is necessary for alleviating oxidative stress induced intestinal epithelial barrier damage and mitochondrial energy metabolism dysfunction in IPEC-J2.

## 1. Introduction

Weaning stress is extremely serious during the initial post-weaning period, which is accompanied by intestinal injury, diarrhea and even death, impairing the growth performance and anti-disease ability of piglets and causing great economic losses to the pig industry [1,2,3]. Moreover, our previous studies and others reported that weaning impaired free-radical metabolism and the antioxidative system and resulted in serious oxidative stress [4,5,6]. Oxidative stress was interpreted as an imbalance between oxidation and anti-oxidation, resulting in the accumulation of reactive oxygen species (ROS) [7]. Several studies reported that imbalance redox biology and oxidative stress led to intestinal function injury [8,9]. However, much uncertainty still exists about the underlying mechanism between oxidative stress and intestinal function.

Intestinal homeostasis depends on the balance between proliferation and cell death of intestinal epithelial cells. The proliferation of intestinal epithelial cells needs a large amount of ATP provided by mitochondria [10], whereas mitochondrial energy metabolism and mitochondrial signaling are associated with ATP and ROS production. Meanwhile, when oxidative stress and antioxidant system are impaired, overproduction ROS in the cell cannot be removed in time, which will cause excessive accumulation of ROS in the cell and further promote mitochondrial oxidative damage [11]. Mitochondrial oxidative damage directly and indirectly impairs the activity of the electron transport chain and energy metabolic enzymes and even leads to cell apoptosis and death [12]. To prevent the damaged mitochondria leading to cell death, the cell initiated a protective process, mitophagy. It is known as the degradation and clearance of dysfunctional mitochondria through the autophagic process [13]. Little data are available regarding the relationship between oxidative stress, intestinal barrier function, mitochondria energy metabolism and mitophagy in the potential mechanisms within intestinal porcine epithelial cells. Adenosine monophosphate-activated protein kinase (AMPK) is in charge of numerous energy metabolism signaling pathways to maintain appropriate intracellular adenosine triphosphate levels in response to cellular stress [14]. Recently, several studies found that AMPK is involved in various aspects of mitochondrial homeostasis [15,16]. Whether AMPK is involved in regulating oxidative stress, mitochondria energy metabolism and mitophagy in intestinal porcine epithelial cells is still unknown.

The present study aimed to determine the effects of H_2_O_2_-induced oxidative stress on intestinal epithelial cell oxidation balance, intestinal epithelial barrier function and mitochondrial energy metabolism and its underlying mechanism. The connection between the intestinal barrier, mitochondria energy metabolism and mitophagy provides insight into the development of nutritional strategies in the prevention of intestinal oxidative stress.

## 2. Materials and Methods

### 2.1. Experiment Design

The IPEC-J2 cell line was kindly provided by Dr Yin Yulong’s laboratory (Institute of Subtropical Agriculture, Chinese Academy of Sciences, ChangSha, China). IPEC-J2 cells were cultured in DMEM/F12 complete medium and divided into 7 treatment groups: control group; AMPK inhibitor group (compound C, 10 μM); mitophagy inhibitor group (mdivi-1, 1 μM); oxidative stress group; oxidative stress + compound C group; oxidative stress group + mdivi-1; oxidative stress + compound C + mdivi-1. The oxidative stress group was simulated by adding 600 μM H_2_O_2_ in the cell culture medium for 8 h and the control group was added with the same amount of PBS. The AMPK inhibitor compound C and mitophagy mdivi-1 inhibitor were added 1 h before adding H_2_O_2_.

### 2.2. MTT Measurement

Using MTT measurement to determine cell viability according to the protocol, as described previously [17]. The cells (10,000/well) were seeded into 96 well plates and were treated with various treatments. After incubation, the absorbance at 570 nm was measured to calculate cell viability.

### 2.3. Antioxidant Enzymes Activities and Malonaldehyde (MDA) Levels

Following the instruction (Nanjing Jiancheng Biotechnology Institute, China), the concentrations of superoxide dismutase (SOD) and catalase (CAT) in IPEC-J2 cells were measured using commercial MDA and GSH kits. The protein concentrations of cells were quantified using the (bicinchoninic acid) BCA Protein Assay Kit. The levels of SOD and CAT were shown as U/mg of protein and the concentrations of MDA were expressed as nmol/mg of protein.

### 2.4. Quantitative Real-Time PCR

Total RNA from cells was extracted using Trizol reagent (Beyotime, Shanghai, China) and the concentrations of the isolated RNA were determined with Nanodrop. cDNA was synthesized by iScript Kit (Bio-Rad, Hercules, CA, USA) and the qRT-PCR reaction utilized the components contained in the SYBR Green Mix (Bio-Rad, Hercules, CA, USA). Samples were normalized by to β-actin and the relative changes of target genes expression were analyzed by 2^−ΔΔCt^ method. The sequences of primers are presented in Table S1.

### 2.5. Western Blot

Total protein from the cells samples was extracted with RIPA lysis buffer (Beyotime, China). Proteins of whole-cell lysates were separated by gel electrophoresis and transferred to a PVDF membrane. The PVDF membranes were sealed with blocking buffer (Beyotime, Shanghai, China) and incubated with primary antibodies overnight at 4 °C and secondary antibodies at room temperature for 1 h. After the chemiluminescent reaction, blots were visualized using an enhanced chemiluminescence detection kit (ECL-Plus, Beyotime, Shanghai, China) with a ChemiScope 3400 (Clinx Science Instruments, Shanghai, China). The western blot was performed by the following antibodies: occludin (HuaBio, Hangzhou, China), claudin-1 (HuaBio, Hangzhou, China), GAPDH (HuaBio, Hangzhou, China) and HRP conjugated anti-rabbit or mouse IgG (HuaBio, Hangzhou, China). In addition, the ZO-1, PINK1, Parkin, LC3, AMPK, phosphorylated-AMPK first antibodies were brought from Abcam (Cambridge, UK). The bands density of protein quantified by Image J.

### 2.6. IPEC-J2 Epithelial Barrier Function Analysis

The IPEC-J2 epithelial barrier function analysis was conducted according to previous reports [17,18]. Briefly, seeding the cells in the Transwell and treated according to the corresponding groups. The trans-epithelium electrical resistant (TER) was recorded by Millicell-ERS epithelial voltohmmeter (Millipore Corp., Billerica, MA, USA). The flux of fluorescein Isothiocyanate-dextran (FD4, Sigma, Northbrook, IL, USA) reflected the IPEC-J2 epithelial barrier function. In addition, the FD4 (final concentration is 500 μg/mL) was added to the upper layer of Transwell for 1.5 h and collected 100 μL cell culture medium to detect the FD4 fluorescence using a fluorescence spectrophotometer (Bio-Tek, Winooski, VT, USA).

### 2.7. MitoSOX

The mitochondria reactive oxygen measurement was conducted according to the manufacture protocol (Invitrogen, Carlsbad, CA, USA). Applied 2.0 mL of 5 μM MitoSOX™ to IPEC-J2 and sealed for 10 min at 37 °C. The MitoSOX™ Red mitochondrial superoxide indicator was measured using Nikon fluorescence microscope (Tokyo, Japan) and BD cytometry (San Jose, CA, USA).

### 2.8. ATP Assay

ATP measurement (Beyotime, Shanghai, China) was conducted following the protocol as described previously [8]. After inhibitors and H_2_O_2_ treatment, the IPEC-J2 cells were collected using 100 μL of ATP releasing reagent from ATP measurement kits. The cells were lysis on ice and centrifuged at 12,000× *g* for 5 min. In addition, the supernatant was further measured the ATP level by a luminometer (Bio-Tek, Winooski, VT, USA) together with 100 μL ATP detect buffer.

### 2.9. Mitochondrial Membrane Potential

Approximately 1 × 10^6^ cells were incubated in 6-well culture dishes and incubated overnight. The IPEC-J2 was rinsed twice by PBS and incubated with JC-1 in the mitochondrial membrane potential assay kit (Beyotime, Shanghai, China) following the protocol. Cells were detected by a fluorescence spectrophotometer (Bio-Tek, Winooski, VT, USA) using 488 nm and 543 nm.

### 2.10. Transmission Electron Microscopy (TEM)

Cell samples were fixed with 2.5% glutaraldehyde and 1% osmium tetroxide. Samples were then dehydrated in acetones, embedded in Spurr resin. Ultra-thin sections were cut and stained with uranyl acetate and lead citrate. The sample block was then sectioned with a LEICA EM UC7 ultratome. Sections were collected on copper grids for imaging under a Hitachi H-7650 electron microscope. Ultrastructural of the mitochondrial were recorded by a transmission electron microscope (Hitachi H-7650, Tokoy, Japan).

### 2.11. The Profiles of Mitochondrial Oxygen Consumption Rate (OCR)

OCR was detected by the Agilent Seahorse XF96 Extracellular Flux Analyzer following the protocols [19,20]. Briefly, 1 × 10^4^ cells were incubated in XF96 plates for 12 h. The cells were washed by phosphate buffer and incubated with Seahorse XF base medium (Agilent, Santa Clara, CA, USA) added with glucose (10 mM; Sigma, Northbrook, IL, USA), glutamax (2 mM; Invitrogen, Carlsbad, CA, USA) and sodium pyruvate (1 mM, Invitrogen, Carlsbad,CA, USA), pH 7.4. OCR was detected by sequential injections of oligomycin A (10 μM; Sigma, Northbrook, IL, USA), FCCP (carbonyl cyanide 4-(trifluoromethoxy) phenylhydrazone, 5 μM; Sigma, Northbrook, IL, USA) and rotenone (15 μM; MilliporeSigma, Northbrook, IL, USA). The cellular basic respiration, cell maximum oxygen consumption, ATP production, cell respiration potential and cell non-mitochondrial respiration were calculated by the mitochondrial oxygen consumption curve.

### 2.12. Cell Transfection and RNA Knockdown

The mitophagy was analyzed using Ad-GFP-LC3 and Ad-HBAD-Mitodsred fluorescence colocalization. Cells were transfected with adenovirus expressing GFP-LC3 (Hanheng, Wuhan, China) at 30 MOI for 24 h and Ad-HBAD-Mitodsred (Hanheng, Wuhan, China) at 100 MOI for 24 h. Ad-GFP-LC3 and Ad-HBAD-Mitodsred fluorescence colocalization were observed under a confocal laser scanning microscope (LSM 880; Carl Zeiss, Oberkochen, Germany).

The RNA knockdown was performed using RNAiMAX (Invitrogen, Carlsbad, CA, USA). THE small interfering RNA (siRNA) was synthesized by GenePharma (Suzhou, China). The sequences for siRNA were as follows: negative control siRNA: 5′-UUCUCCGAACGUGUCACGUTT-3′; Pig PINK1 siRNA: 5′-GCUAACGUGCUUCAUUUAATT-3′.

### 2.13. Measurement of Apoptosis and Necrosis

The measurement of apoptosis and necrosis using staining by annexin-FITC-PI and performed according to manufacture instruction (Beyotime, Shanghai, China). The apoptosis and necrosis cells were quantified by flow cytometry (BD Arial, San Jose, CA, USA).

### 2.14. Statistical Analysis

Data are presented as the mean ± SD. One-way analysis of variance (ANOVA) was determined by Graphpad Prism 7. Significance was established at *p <* 0.05.

## 3. Results

### 3.1. Effects of Different Concentrations of H_2_O_2_ Treatment on AMPK Signaling Pathway and Mitophagy-Related Protein Expression

**Figure 1:**
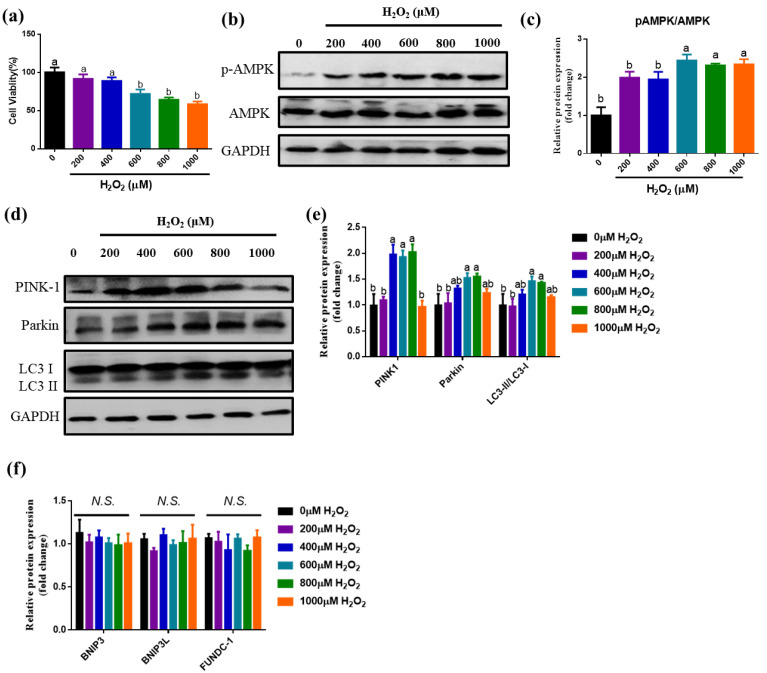
The effect of treated with different concentrations of H_2_O_2_ on AMPK signaling pathway and mitophagy related proteins expression. (**a**) The effects of different levels of H_2_O_2_ on the viability of IPEC-J2; (**b**) Western blot analysis of AMPK expression; (**c**) The levels of AMPK protein after treated with different levels of H_2_O_2_. (**d**) Western blot analysis of PINK1, Parkin and LC3II/I protein expression; (**e**) The relative expression of PINK1, Parkin and LC3II/I protein after treated with different concentrations of H_2_O_2_. (**f**) The relative gene expression of BNIP3, BNIP3L and FUNDC-1 after treated with different concentrations of H_2_O_2_. N.S. means no significantly difference. Data showed as mean ± SD. Values with different letters indicate a significant difference (*p <* 0.05).

To explore the relationship between oxidative stress, mitophagy and AMPK signaling pathways, so we established an H_2_O_2_-induced oxidative stress model of IPEC-J2 cells following the previous data [17]. As shown in Figure 1a, we found that treated cells with 600 μM, 800 μM and 1000 μM H_2_O_2_ for 8 h significantly decreased cell viability (*p <* 0.05). Therefore, in this study, 600 μM H_2_O_2_ was used to induce an IPEC-J2 oxidative stress model. In addition, in comparison with the control, 600 μM H_2_O_2_ treatment dramatically enhanced p-AMPK protein level (*p <* 0.05) (Figure 1b,c). Moreover, 600 μM and 800 μM H_2_O_2_ treatments enhanced the levels of PINK1, Parkin and the LC3II/I ratio (*p* *<* 0.05), while it did not influence the expression of BNIP3, BNIP3L and FUNDC-1 (*p* *>* 0.05) (Figure 1d–f). These results indicated that H_2_O_2_-induced oxidative stress promoted AMPK phosphorylation and PINK1-Parkin mediation mitophagy.

### 3.2. Effects of Inhibited AMPK and Mitophagy on the Oxidative Stress Response of IPEC-J2 in H_2_O_2_-Induced Oxidative Stress Model

**Figure 2:**
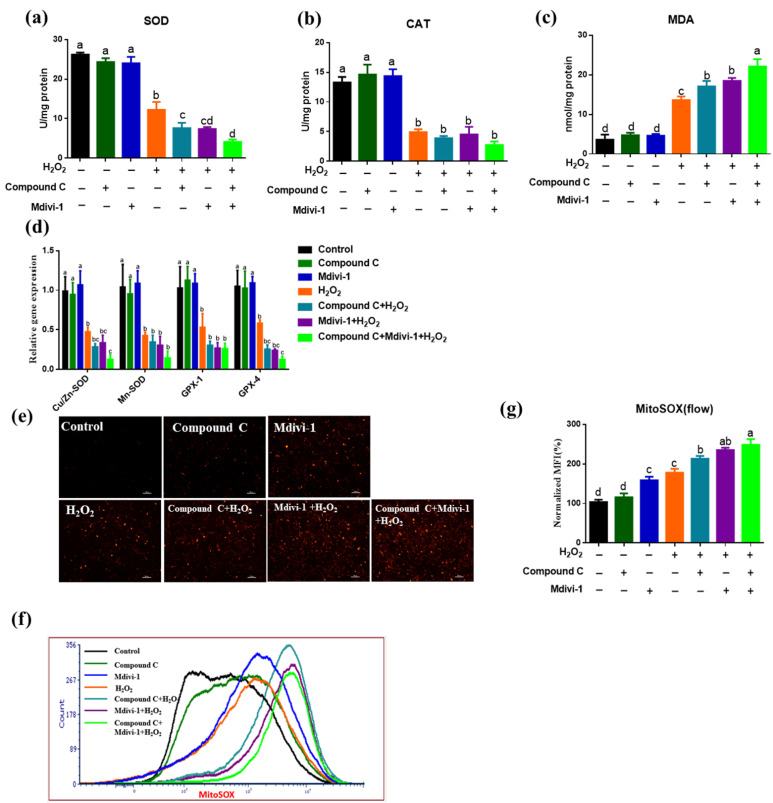
The effect of inhibiting AMPK and mitophagy on H_2_O_2_-induced oxidative stress in intestinal epithelial cells. (**a**) In the oxidative stress model induced by H_2_O_2_, the effect of inhibiting AMPK signaling pathway and mitophagy on SOD activity; (**b**) Inhibiting the impact of AMPK signaling pathway and mitophagy on CAT activity; (**c**) The effect of inhibiting AMPK signaling pathway and mitophagy on MDA levels; (**d**) Inhibiting AMPK signaling pathway and mitophagy on the levels of *Cu/Zn-SOD, Mn-SOD, GPX-1, GPX-4*; (**e**) MitoSOX fluorescence staining; (**f**) MitoSOX fluorescence intensity detected by BD flow cytometry; (**g**) MitoSOX statistics quantified. Data showed as mean ± SD. Values with different letters indicate a significant difference (*p <* 0.05).

To detect the function of AMPK and mitophagy in H_2_O_2_ induced oxidative stress, we added AMPK inhibitor (compound C) and mitophagy inhibitor (mdivi-1) in the oxidative stress model. The data indicated that H_2_O_2_ significantly reduced the SOD and CAT activities of intestinal epithelial cells than the control group (*p <* 0.05) (Figure 2a,b) and increased the MDA content (*p <* 0.05) (Figure 2c). Moreover, oxidative stress significantly reduced the expression of *Cu/Zn-SOD*, *Mn-SOD*, *GPX-1*, *GPX-4* (*p <* 0.05) (Figure 2d). Compared with the oxidative stress group, compound C and mdivi-1 significantly reduced the activity of SOD in H_2_O_2_ treated cells (*p <* 0.05) (Figure 2a), but no differences were found on CAT activity, MDA content and the anti-oxidative stress-related genes expression (*p* *>* 0.05) (Figure 2b–d). Furthermore, inhibition of AMPK and mitophagy at the same time aggravated the reduction of SOD activity induced by H_2_O_2_ (*p <* 0.05) (Figure 2a). As presented in Figure 2e–g, the oxidative stress group enhanced the mitochondrial ROS level than the control group (*p <* 0.05). Compared with the oxidative stress group, compound C + H_2_O_2_ group, mdivi-1+ H_2_O_2_ group and compound C +mdivi-1+ H_2_O_2_ group had a higher mitochondrial ROS level (*p <* 0.05). These data revealed that inhibiting the AMPK and mitophagy in the oxidative stress model led to the accumulation of mitochondrial ROS in the cells.

### 3.3. Effects of Inhibition of AMPK and Mitophagy on the IPEC-J2 Epithelial Barrier Function in H_2_O_2_-Oxidative Stress Model

**Figure 3:**
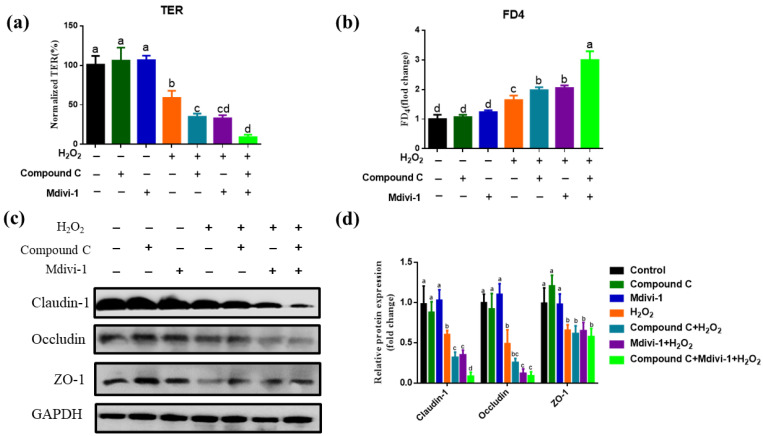
The effects of inhibiting AMPK and mitophagy on intestinal barrier function in H_2_O_2_-induced oxidative stress model of IPEC-J2. (**a**) the effect of inhibiting AMPK and mitophagy on TER; (**b**) the effect of inhibiting AMPK signal pathway and mitophagy on FD4 flux; (**c**) Western analysis of occludin, claudin-1 and zo-1 protein expression; (**d**) Relative protein levels of occludin, claudin-1 and zo-1; Data expressed as mean ± SD. Values with different letters indicate a significant difference (*p <* 0.05).

H_2_O_2_ induced oxidative stress led to intestinal epithelial barrier dysfunction. H_2_O_2_ treatment significantly reduced TER and enhanced FD4 flux than the control group (Figure 3 a,b). Moreover, H_2_O_2_ treatment had a lower tight junction proteins expression than the control group (*p <* 0.05) (Figure 3c,d). Compared with the oxidative stress group, compound C + H_2_O_2_ and mdivi-1+ H_2_O_2_ treatment dramatically decreased the TER and enhanced the permeability of FD4 in intestinal epithelial cells (*p* *<* 0.05) (Figure 3a,b). In comparison with the H_2_O_2_ group, compound C + mdivi-1 + H_2_O_2_ treatment aggravated the intestinal barrier damage caused by H_2_O_2_ (*p* *<* 0.05). These results together revealed that inhibiting the AMPK and the mitophagy aggravate IPEC-J2 barrier dysfunction induced by H_2_O_2_ in IPEC-2.

### 3.4. Effects of Inhibition of AMPK and Mitophagy on the Mitochondrial Function and Ultrastructure of IPEC-J2 in H_2_O_2_-Induced Oxidative Stress Model

**Figure 4:**
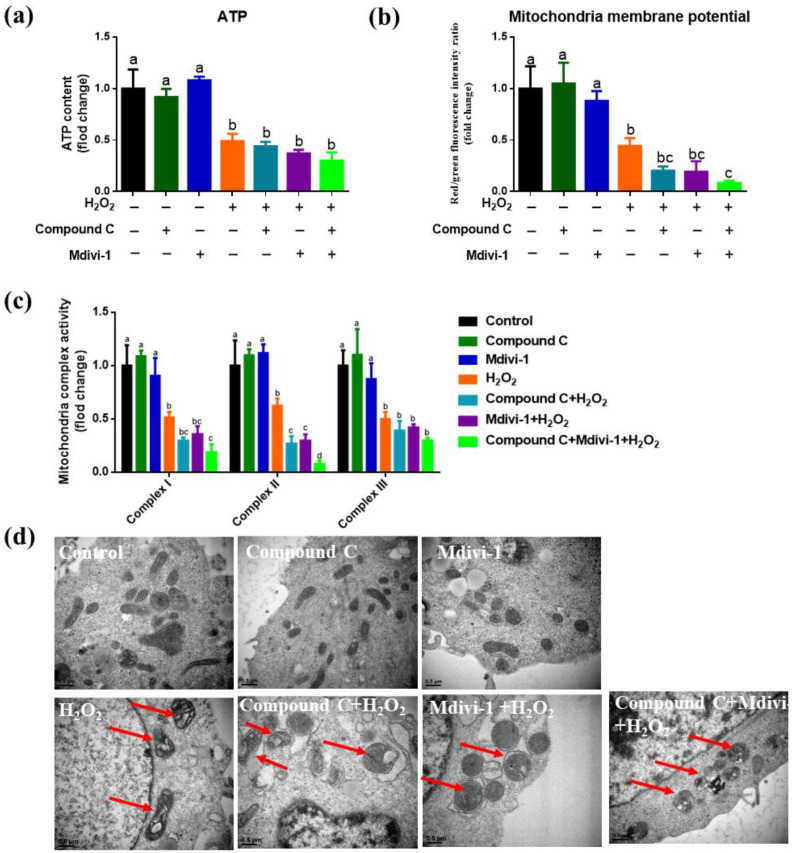
The effect of inhibiting AMPK and mitophagy on mitochondrial function in H_2_O_2_-induced oxidative stress model of IPEC-J2. (**a**) The effect of inhibiting AMPK and mitophagy on ATP production; (**b**) the effect of inhibiting AMPK and mitophagy on mitochondrial membrane potential; (**c**) the effect of inhibiting AMPK and mitophagy on the activity of mitochondrial respiratory chain complexes I, II, III. (**d**) Mitochondrial ultrastructure detected by TEM. Data expressed as mean ± SD. ^a,b,c,d^ Means with different letters differ significantly (*p <* 0.05).

In comparison with the control IPEC-J2, H_2_O_2_ significantly reduced ATP production, mitochondrial membrane potential and mitochondrial respiratory chain complexes I, II, III activity in cells (*p <* 0.05) (Figure 4a–c). Compared with the oxidative stress group, added compound C and mdivi-1 significantly reduced the activity of mitochondrial respiratory chain complex II (*p <* 0.05) (Figure 4c), while added compound C and mdivi-1 in oxidative stress did not influence the ATP production, mitochondrial membrane potential and complex I, III (*p >* 0.05) (Figure 4a–c). In comparison with the H_2_O_2_ treated IPEC-J2, compound C + mdivi-1 + H_2_O_2_ treatment significantly reduced the mitochondrial membrane potential and the activities of mitochondrial respiratory chain complex I, II (*p <* 0.05) (Figure 4a–c). Compared with the control group, we can observe obvious mitochondrial swelling, respiratory cristae breakage and mitochondrial vacuolation in the oxidative stress group, compound C + H_2_O_2_ group, mdivi-1+ H_2_O_2_ group, compound C +mdivi-1+ H_2_O_2_ group.

As shown in Figure S1, we indicated that compared with the control group, compound C, mdivi-1, compound C + H_2_O_2_, mdivi-1+ H_2_O_2_ and compound C + mdivi-1+ H_2_O_2_ group enhanced the ratio of necrosis or apoptosis cells (*p <* 0.05). Compared with the oxidative stress group, mdivi-1+ H_2_O_2_ and compound C + mdivi-1+ H_2_O_2_ group significantly increased the apoptosis cells level and compound C + mdivi-1+ H_2_O_2_ treatment dramatically enhanced the necrosis cells ratio (*p <* 0.05) (Figure S1).

### 3.5. Effects of Inhibition of AMPK and Mitophagy on the Mitochondrial Metabolism of IPEC-J2 in H_2_O_2_-Induced Oxidative Stress Model

**Figure 5:**
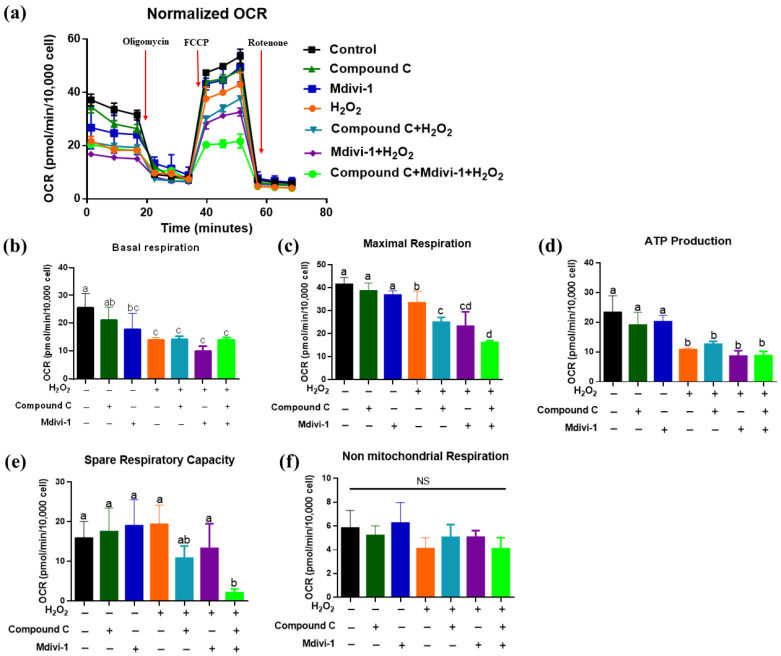
The effect of inhibiting AMPK and mitophagy on mitochondrial energy metabolism in H_2_O_2_-induced oxidative stress model of IPEC-J2. (**a**) In the H_2_O_2_-induced IPEC-J2 cell oxidative stress model, the effect of inhibiting AMPK signaling pathway and mitophagy on cell oxygen consumption rate (normalized to baseline); (**b**) The effects of inhibiting of AMPK and mitophagy on cellular basic respiration (**c**) The effect of inhibition of AMPK and mitophagy on cell maximum oxygen consumption; (**d**) The effects of inhibition of AMPK and mitophagy on cell ATP production; (**e**) The effects of inhibition of AMPK and mitophagy on spare respiration capacity; (**f**) The effect of inhibition of AMPK and mitophagy on cell non-mitochondrial respiration. Data presented as mean ± SD. Values with different letters indicate a significant difference (*p <* 0.05).

The OCR data show that in comparison with the control group, we found the lower oxygen consumption rate of IPEC-J2 in the oxidative stress group, compound C + H_2_O_2_ group, mdivi-1+ H_2_O_2_ group, compound C +mdivi-1+ H_2_O_2_ group (Figure 5a). Moreover, H_2_O_2_ treatment significantly decreased basal respiration, maximal respiration and ATP production than the control group (*p* *<* 0.05) (Figure 5b–d). Compared with the oxidative stress group, treated with compound C and mdivi-1 significantly reduced the maximum respiratory oxygen consumption of IPEC-J2 (*p <* 0.05) (Figure 5c). In comparison with the H_2_O_2_ group, compound C +mdivi-1+ H_2_O_2_ treatment significantly reduced maximum respiratory oxygen consumption and spare respiratory capacity of IPEC-J2 (*p <* 0.05) (Figure 5e). The non-mitochondrial respiratory capacity was not influenced by different treatments (*p* *>* 0.05) (Figure 5f). These results demonstrated that simultaneous inhibition of AMPK and mitophagy significantly impaired the spare respiratory capacity of IPEC-J2 and aggravated the decrease in the maximum respiratory oxygen consumption of cells caused by H_2_O_2_.

### 3.6. Effects of Inhibition of AMPK and Mitophagy on the Mitophagy Level of IPEC-J2 in H_2_O_2_-Induced Oxidative Stress Model

**Figure 6:**
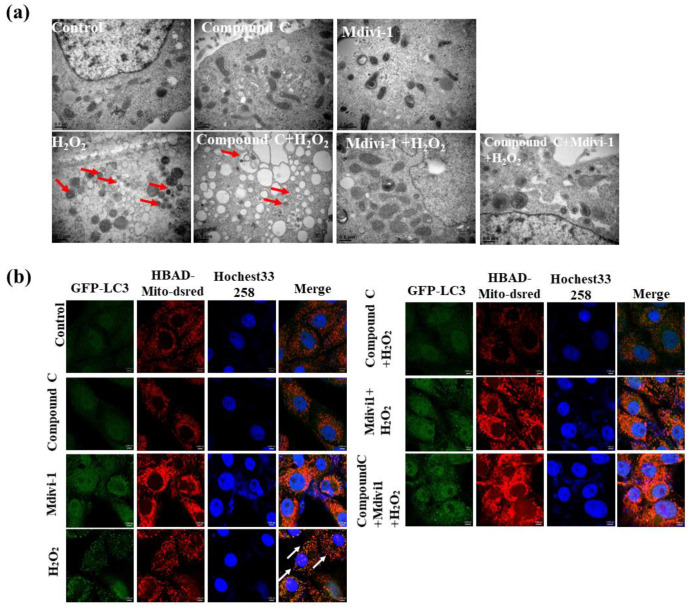
The effect of inhibiting AMPK and mitophagy on the mitochondrial autophagosomes and Co-localization of autophagic vesicles and mitochondria in the H_2_O_2_ treated IPEC-J2 cells. (**a**) Mitochondrial autophagosomes ultrastructure (scale bar: 0.5 μm). (**b**) Colocalization of autophagic vesicles (GFP-LC3) and mitochondria (HBAD-Mito-dsred) (scale bar: 5000 nm). Data showed as mean ± SD.

TEM detected the ultrastructure of mitochondrial autophagosome in IPEC-J2. The damaged mitochondria were wrapped by the double-membrane autophagic vesicles in the oxidative stress group and compound C + H_2_O_2_ group (Figure 6a). Compared with the oxidative stress group, the number of mitochondrial autophagosomes significantly reduced and mitochondria were swollen in the mdivi-1+ H_2_O_2_ group and compound C + mdivi-1+ H_2_O_2_ group. Furthermore, confocal microscopy showed that H_2_O_2_ treatment dramatically enhanced the colocalization of LC3 and Mito in IPEC-J2 (Figure 6b), while the colocalization was significantly reduced in the compound C + H_2_O_2_ group, mdivi-1+ H_2_O_2_ and compound C +mdivi-1+ H_2_O_2_ (Figure 6b). These data suggested that the AMPK is vital for oxidative stress induced protective mitophagy.

### 3.7. Effects of Knockdown of AMPK, PINK1 and Parkin in H_2_O_2_-Induced Oxidative Stress Model

Our previous data indicated that knockdown of PRKAA1 (encoding AMPK α) and Parkin aggravate H_2_O_2_ induced oxidative stress, intestinal barrier disruption and mitochondrial impairment [17]. As shown in Figure S2, we found that siPINK1 treatment significantly declined the *Cu/Zn-SOD* expression and enhanced MDA level in H_2_O_2_-induced oxidative stress model (*p <* 0.05) (Figure S2a,c). Moreover, knockdown of PINK1 decreased ZO-1 protein level and ATP content in H_2_O_2_ treated cells (*p <* 0.05) (Figure S2d,e,g).

## 4. Discussion

H_2_O_2_ is both an exogenous and endogenous pro-oxidative mediator that can lead to lipid peroxidation within the cell membranes and destroy the structure and function of the cell membrane, so it has been utilized in establishing an oxidative stress model in vitro [21,22] and in vivo [23]. The results showed that treated IPEC-J2 with 600 μM, 800 μM and 1000 μM H_2_O_2_ for 8 h significantly reduced cell viability and 600 μM H_2_O_2_ was used in this study to set an oxidative stress model. Chen et al. (2018) found that treated IPEC-J2 with 0.75 mM H_2_O_2_ for 4 h significantly reduced cell viability [24]. Yuan et al. (2019) found that treatment of IPEC-J2 cells with 0.5 mM H_2_O_2_ for 6 h significantly inhibited cell growth [25]. AMPK is a serine/threonine-protein kinase that acts as an energy and redox balance sensor in cells and is vital for the upregulation of catabolism [26]. Kaspar et al. (2009) have shown that AMPK activation could alleviate oxidative stress and promotes cell survival through regulating the expression of antioxidant-related genes [27]. We found that 600 μM H_2_O_2_ treatment dramatically increased the expression of *p*-AMPK. Similarly, He et al. (2020) also indicated that H_2_O_2_ treatment significantly increased AMPK activity [28]. Moreover, mitophagy has been reported that involved in alleviating oxidative stress and protecting mitochondria integrity in aging [12], cardiac ischemia-reperfusion injury [29] and neurodegeneration [30]. Furthermore, our previous data reported that PINK1/Parkin mediated mitophagy plays a vital role in regulating intestine disruption induced by oxidative stress in piglets [8]. PINK1 accumulated on mitochondrial upon loss of the mitochondrial membrane potential and then PINK1 in turn recruits phosphorylates Parkin, an E3 ubiquitin ligase that marks mitochondria for degradation by mitophagy [31]. Currently, we found that H_2_O_2_ significantly promoted mitophagy, indicated by increased levels of PINK1, Parkin and the LC3II/I ratio in cells treated with H_2_O_2_. Consistently, Wang et al. (2020) showed that cadmium induces oxidative stress promoted mitophagy via AMP-activated protein kinases activation in a PINK1/Parkin-dependent manner in PC12 cells [32].

Increasing pieces of evidence pointed to a specific regulation on mitochondrial homeostasis by AMPK [15,33]. However, the effects of AMPK on mitophagy in oxidative stress remained unclear. The data indicated that H_2_O_2_ treatment significantly reduced the SOD, CAT activities and increased the MDA content of IPEC-J2. Moreover, AMPK signaling pathway inhibitor compound C and mitophagy inhibitor mdivi-1 aggravated the oxidative stress in IPEC-J2. Moreover, this experiment also found that in the oxidative stress model, inhibited the AMPK and the mitophagy will lead to the accumulation of mitochondrial ROS in the IPEC-J2. In line with our finding, Wu et al. (2018) also found that inhibited AMPK signaling pathway led to an enhancement in ROS in HT22 cells treated with H_2_O_2_ [34].

The intestinal epithelial layer has unique organizational capabilities, which can achieve rapid self-renewal through the process of crypt stem cell proliferation and crypt-to-villi cell differentiation [35]. Oxidative stress in the intestinal mucosa will disrupt the self-renewal process of the intestine and redox signaling contributes to the development of intestinal degenerative diseases, like inflammation and cancer [36,37]. Currently, the data indicated that H_2_O_2_ destroyed the IPEC-J2 epithelial barrier function, manifested by reduced the TER and enhanced FD4 permeability, which is consistent with previous reports [28]. Tight junction proteins are involved in the selective permeability between intestinal cells [38]. Our results revealed that H_2_O_2_ reduced the levels of tight junction proteins and inhibited AMPK, mitophagy exacerbated of H_2_O_2_-induced intestinal epithelial barrier injury. Several studies have reported that AMPK signaling pathway is involved in mediating barrier function. Wu et al. (2018) reported that metformin alleviates LPS-induced intestinal epithelial injury via activating AMPK [39]. Scharl et al. (2009) showed that the treatment of T84 with IFN-γ reduced TER, intestinal epithelial permeability and declined the levels of tight junction proteins and activates the AMPK [40].

Mitochondrial DNA is vulnerable to ROS attack because mtDNA is closed to the electron transport chain and lacked the protection of histones. Moreover, mtDNA encodes a variety of proteins, including enzymes on the mitochondrial respiratory chain, damaged mtDNA may cause energy damage, thereby exacerbating oxidative stress. Our data indicated that H_2_O_2_-induced oxidative stress may lead to cell mitochondrial energy metabolism disorder and mitochondrial respiratory chain complexes impaired and this process is regulated by the AMPK signaling pathway and mitophagy. We found that oxidative stress significantly reduced intestinal epithelial cell ATP production, mitochondrial membrane potential and mitochondrial respiratory chain complexes activity. Inhibiting AMPK and mitophagy aggravated the decline of mitochondrial membrane potential and mitochondrial respiratory chain complex I and II activity caused by H_2_O_2_. Consistently, He et al. (2020) indicated that the H_2_O_2_ led to the decreased mitochondrial membrane potential and mtDNA copy number of IPEC-J2, which was regulated by the AMPK signal pathway [28].

Intestinal homeostasis and renewal rely on ATP provided by cell energy metabolism. In this study, we utilized the XF96 seahorse energy analyzer to detect cell energy metabolism. OCR can reflect the respiration rate of IPEC-J2 mitochondria. Oligomycin can bind to the complex V in the oxidative respiratory chain to inhibit ATP synthesis and then reduce the mitochondrial respiration associated with the synthesis of cellular ATP and reduce the OCR value. FCCP is an uncoupling agent, utilized to detect the maximal respiration and spare respiratory capacity, which can reflect the ability of cells to respond to enhanced energy demand or stress [41]. Non-mitochondrial oxygen consumption inhibits mitochondrial metabolism through the mitochondrial electronic respiratory chain complex I inhibitor rotenone. In this study, we found the lower oxygen consumption of IPEC-J2 in the oxidative stress group, compound C + H_2_O_2_ group, mdivi-1+ H_2_O_2_ group, compound C + mdivi-1+ H_2_O_2_ group, in comparison with the control group. This finding was like He et al. (2020), who found that H_2_O_2_ treatment can lead to a decrease in ATP production, basal respiratory oxygen consumption, maximum respiratory oxygen consumption and spare respiratory capacity [28]. Auciello et al. (2014) found that oxidative stress mainly activates AMPK by enhancing cellular AMP and/or ADP, thereby promoting intracellular energy metabolism [42]. Moreover, we found that compound C +mdivi-1+ H_2_O_2_ treatment significantly reduced the cell’s maximum respiratory oxygen consumption and spare respiratory capacity. Taken together, these results suggested that inhibited AMPK and mitophagy led to the impaired anti-stress ability of IPEC-J2. In line with Zhang et al. (2019), who found that in a model of oxidative stress caused by myocardial ischemia-reperfusion injury, melatonin relieves myocardial injury by promoting mitochondrial fusion and mitophagy and activating the AMPK-OPA1 signaling pathway [43].

In response to oxidative stress and impaired mitochondria, cells initiated mitophagy to prevent cell apoptosis and death. Mitophagy is promoted by ROS overaccumulation, depolarized mitochondria and energy metabolism dysfunction [44]. Upon mitochondrial damage such as depolarization, PINK1 accumulates on the outer mitochondrial membrane and can catalyze the ubiquitin and the ubiquitin-like domain of Parkin [45]. These results demonstrated, unequivocally, that compound C and mdivi-1 significantly inhibited the protective mitophagy response induced by oxidative stress, indicated by mitochondrial autophagosomes number. To directly visualize the activation of mitophagy in IPEC-J2 cells, we used the Ad-GFP-LC3 and Ad-HBAD-Mito-dsred to monitor the formation of mitophagosomes. The confocal microscopy showed that the co-localization of Ad-GFP-LC3 and Ad-HBAD-Mito-dsred was significantly reduced after inhibiting AMPK and mitophagy. These data suggested that the AMPK signal pathway is vital for oxidative stress induced protective mitophagy. Consistent with our findings, Laker et al. (2017) reported that AMPK phosphorylation of Ulk1 is required for targeting mitochondria to lysosomes in exercise-induced mitophagy [16].

Studies showed that AMPK plays a role in mitophagy and mitochondrial removal in response to cellular stress [46,47]. Seabright et al. (2020) AMPK activation induces mitophagy and promotes mitochondrial fission while activating TBK1 in a PINK1-Parkin independent manner [48]. Wang et al. (2018) showed that AMPK protects against the development of heart failure by enhancing mitophagy via PINK1 phosphorylation [49]. Moreover, they indicated that Ala mutation of PINK1 at Ser495 partially suppressed AMPK overexpression-induced mitophagy and improvement of mitochondrial function in phenylephrine-stimulated cardiomyocytes [49]. Zhang et al. (2020) reported that myocardial ischemia-reperfusion injury (known as oxidative stress condition) via improving mitochondrial fusion/mitophagy and activating the AMPK-OPA1 signaling pathways [43] Wang et al. (2014) reported that AMPK may mediate paraquat-induced myocardial anomalies possibly by regulating the AMPK/mTOR-dependent autophagy [50]. Studies indicated that mammalian STE20-Like Kinase 1 deletion alleviates renal ischemia-reperfusion injury and non-alcoholic fatty liver disease via modulating mitophagy and the AMPK signaling pathway [51,52]. Diquat is a well-established piglet’s intestinal oxidative stress model [53,54,55,56]. Our previous studies indicated that diquat induced oxidative stress increases intestinal permeability, impairs mitochondrial function and triggers mitophagy and AMPK in piglets [8,17,57].

## 5. Conclusions

In summary, we show that H_2_O_2_ induced oxidative stress caused intestinal epithelial barrier damage and mitochondrial energy metabolism disorder and activated PINK1-Parkin-mediated mitophagy and AMPK signaling pathways. Furthermore, inhibited AMPK and mitophagy aggravated H_2_O_2_-induced oxidative stress, IPEC-J2 barrier injury and mitochondrial energy metabolism dysfunction, suggesting that AMPK and mitophagy played important role in oxidative stress-induced intestinal damage regulation.

## Data Availability

Data is contained within the article and Supplementary Materials.

## Supplementary Materials

The following are available online at https://www.mdpi.com/article/10.3390/antiox10122010/s1, Figure S1: The effect of inhibiting AMPK and mitophagy on the ratio of apoptosis and necrosis cells in H2O2-induced oxidative stress model of intestinal epithelial cells, Figure S2: The effects of knockdown of PINK1 in the H_2_O_2_-induced oxidative stress model of intestinal epithelial cells. Table S1: Primer sequences used for real-time PCR.

## References

[1] Smith F., Clark J.E., Overman B.L., Tozel C.C., Huang J.H., Rivier J.E., Blikslager A.T., Moeser A.J. (2010). Early weaning stress impairs development of mucosal barrier function in the porcine intestine. Am. J. Physiol. Gastrointest. Liver Physiol..

[2] Wijtten P.J., Van Der Meulen J., Verstegen M.W. (2011). Intestinal barrier function and absorption in pigs after weaning: A review. Br. J. Nutr..

[3] Wang J., Xiao Y., Li J., Qi M., Tan B. (2021). Serum biochemical parameters and amino acids metabolism are altered in piglets by early-weaning and proline and putrescine supplementations. Anim. Nutr..

[4] Luo Z., Zhu W., Guo Q., Luo W., Zhang J., Xu W., Xu J. (2016). Weaning Induced Hepatic Oxidative Stress, Apoptosis, and Aminotransferases through MAPK Signaling Pathways in Piglets. Oxid. Med. Cell. Longev..

[5] Yin J., Wu M.M., Xiao H., Ren W.K., Duan J.L., Yang G., Li T.J., Yin Y.L. (2014). Development of an antioxidant system after early weaning in piglets2. J. Anim. Sci..

[6] Cao S.T., Wang C.C., Wu H., Zhang Q.H., Jiao L.F., Hu C.H. (2018). Weaning disrupts intestinal antioxidant status, impairs intestinal barrier and mitochondrial function, and triggers mitophagy in piglets1. J. Anim. Sci..

[7] Birben E., Sahiner U.M., Sackesen C., Erzurum S., Kalayci O. (2012). Oxidative Stress and Antioxidant Defense. World Allergy Organ. J..

[8] Cao S.T., Wu H., Wang C.C., Zhang Q.H., Jiao L.F., Lin F.H., Hu C.H.H. (2018). Diquat-induced oxidative stress increases intestinal permeability, impairs mitochondrial function, and triggers mitophagy in piglets1. J. Anim. Sci..

[9] Li M., Yuan D., Liu Y., Jin H., Tan B. (2020). Dietary Puerarin Supplementation Alleviates Oxidative Stress in the Small Intestines of Diquat-Challenged Piglets. Animals.

[10] Rath E., Moschetta A., Haller D. (2018). Mitochondrial function—Gatekeeper of intestinal epithelial cell homeostasis. Nat. Rev. Gastroenterol. Hepatol..

[11] Peoples J.N., Saraf A., Ghazal N., Pham T.T., Kwong J.Q. (2019). Mitochondrial dysfunction and oxidative stress in heart disease. Exp. Mol. Med..

[12] Wu N.N., Zhang Y., Ren J. (2019). Mitophagy, Mitochondrial Dynamics, and Homeostasis in Cardiovascular Aging. Oxidative Med. Cell. Longev..

[13] Baechler B.L., Bloemberg D., Quadrilatero J. (2019). Mitophagy regulates mitochondrial network signaling, oxidative stress, and apoptosis during myoblast differentiation. Autophagy.

[14] Wu S., Zou M.-H. (2020). AMPK, Mitochondrial Function, and Cardiovascular Disease. Int. J. Mol. Sci..

[15] Herzig S., Shaw R.J. (2018). AMPK: Guardian of metabolism and mitochondrial homeostasis. Nat. Rev. Mol. Cell Biol..

[16] Laker R.C., Drake J.C., Wilson R.J., Lira V.A., Lewellen B.M., Ryall K.A., Fisher C.C., Zhang M., Saucerman J.J., Goodyear L.J. (2017). Ampk phosphorylation of Ulk1 is required for targeting of mitochondria to lysosomes in exercise-induced mitophagy. Nat. Commun..

[17] Cao S., Wang C., Yan J., Li X., Wen J., Hu C. (2020). Curcumin ameliorates oxidative stress-induced intestinal barrier injury and mitochondrial damage by promoting Parkin dependent mitophagy through AMPK-TFEB signal pathway. Free. Radic. Biol. Med..

[18] Xiao K., Cao S., Jiao L., Song Z., Lu J., Hu C. (2017). TGF-β1 protects intestinal integrity and influences Smads and MAPK signal pathways in IPEC-J2 after TNF-α challenge. Innate Immun..

[19] Lin H.H., Chung Y., Cheng C.-T., Ouyang C., Fu Y., Kuo C.-Y., Chi K.K., Sadeghi M., Chu P., Kung H.J. (2018). Autophagic reliance promotes metabolic reprogramming in oncogenic KRAS-driven tumorigenesis. Autophagy.

[20] Tan B., Xiao H., Li F., Zeng L., Yin Y. (2015). The profiles of mitochondrial respiration and glycolysis using extracellular flux analysis in porcine enterocyte IPEC-J2. Anim. Nutr..

[21] Zou Y., Wang J., Peng J., Wei H. (2016). Oregano Essential Oil Induces SOD1 and GSH Expression through Nrf2 Activation and Alleviates Hydrogen Peroxide-Induced Oxidative Damage in IPEC-J2 Cells. Oxid. Med. Cell. Longev..

[22] Yin J., Wu M., Li Y., Ren W., Xiao H., Chen S., Li C., Tan B., Ni H., Xiong X. (2017). Toxicity assessment of hydrogen peroxide on Toll-like receptor system, apoptosis, and mitochondrial respiration in piglets and IPEC-J2 cells. Oncotarget.

[23] Duan J., Yin J., Ren W., Liu T., Cui Z., Huang X., Wu L., Kim S.W., Liu G., Wu X. (2016). Dietary supplementation with l-glutamate and l-aspartate alleviates oxidative stress in weaned piglets challenged with hydrogen peroxide. Amino Acids.

[24] Chen Z., Yuan Q., Xu G., Chen H., Lei H., Su J. (2018). Effects of Quercetin on Proliferation and H_2_O_2_-Induced Apoptosis of Intestinal Porcine Enterocyte Cells. Mol..

[25] Yuan Z., Liang Z., Yi J., Chen X., Li R., Wu Y., Wu J., Sun Z. (2019). Protective Effect of Koumine, an Alkaloid from Gelsemium Sempervirens, on Injury Induced by H2O2 in IPEC-J2 Cells. Int. J. Mol. Sci..

[26] Li Y., Chen Y. (2019). AMPK and Autophagy. Adv. Exp. Med. Biol..

[27] Kaspar J.W., Niture S.K., Jaiswal A.K. (2009). Nrf2:INrf2 (Keap1) signaling in oxidative stress. Free. Radic. Biol. Med..

[28] He L., Long J., Zhou X., Liu Y., Li T., Wu X. (2020). Serine is required for the maintenance of redox balance and proliferation in the intestine under oxidative stress. FASEB J..

[29] Yang M., Linn B.S., Zhang Y., Ren J. (2019). Mitophagy and mitochondrial integrity in cardiac ischemia-reperfusion injury. Biochim. Biophys. Acta. Mol. Basis Dis..

[30] Barodia S.K., Creed R.B., Goldberg M.S. (2017). Parkin and PINK1 functions in oxidative stress and neurodegeneration. Brain Res. Bull..

[31] Bader V., Winklhofer K.F. (2020). PINK1 and Parkin: Team players in stress-induced mitophagy. Biol. Chem..

[32] Wang T., Zhu Q., Cao B., Yuan Y., Wen S., Liu Z. (2020). Cadmium induces mitophagy via AMP-activated protein kinases activation in a PINK1/Parkin-dependent manner in PC12 cells. Cell Prolif..

[33] Mottillo E.P., Desjardins E.M., Crane J.D., Smith B.K., Green A.E., Ducommun S., Henriksen T.I., Rebalka I.A., Razi A., Sakamoto K. (2016). Lack of Adipocyte AMPK Exacerbates Insulin Resistance and Hepatic Steatosis through Brown and Beige Adipose Tissue Function. Cell Metab..

[34] Wu X., Luo P., Rao W., Dai S., Zhang L., Ma W., Pu J., Yu Y., Wang J., Fei Z. (2018). Homer1a Attenuates Hydrogen Peroxide-Induced Oxidative Damage in HT-22 Cells through AMPK-Dependent Autophagy. Front. Neurosci..

[35] Bankaitis E.D., Ha A., Kuo C.J., Magness S.T. (2018). Reserve Stem Cells in Intestinal Homeostasis and Injury. Gastroenterology.

[36] Mariani F., Sena P., Roncucci L. (2014). Inflammatory pathways in the early steps of colorectal cancer development. World J. Gastroenterol..

[37] Bhattacharyya A., Chattopadhyay R., Mitra S., Crowe S.E. (2014). Oxidative Stress: An Essential Factor in the Pathogenesis of Gastrointestinal Mucosal Diseases. Physiol. Rev..

[38] Camilleri M., Madsen K., Spiller R., Meerveld B.G.-V., Verne G.N. (2012). Intestinal barrier function in health and gastrointestinal disease. Neurogastroenterol. Motil..

[39] Wu W., Wang S., Liu Q., Shan T., Wang Y. (2018). Metformin Protects against LPS-Induced Intestinal Barrier Dysfunction by Activating AMPK Pathway. Mol. Pharm..

[40] Scharl M., Paul G., Barrett K.E., McCole D.F. (2009). AMP-activated Protein Kinase Mediates the Interferon-γ-induced Decrease in Intestinal Epithelial Barrier Function. J. Biol. Chem..

[41] Sun S., Hu F., Wu J., Zhang S. (2017). Cannabidiol attenuates OGD/R-induced damage by enhancing mitochondrial bioenergetics and modulating glucose metabolism via pentose-phosphate pathway in hippocampal neurons. Redox Biol..

[42] Auciello F.R., Ross F.A., Ikematsu N., Hardie D.G. (2014). Oxidative stress activates AMPK in cultured cells primarily by increasing cellular AMP and/or ADP. FEBS Lett..

[43] Zhang Y., Wang Y., Xu J., Tian F., Hu S., Chen Y., Fu Z. (2019). Melatonin attenuates myocardial ischemia-reperfusion injury via improving mitochondrial fusion/mitophagy and activating the AMPK-OPA1 signaling pathways. J. Pineal Res..

[44] Wauters F., Cornelissen T., Imberechts D., Martin S., Koentjoro B., Sue C., Vangheluwe P., Vandenberghe W. (2020). LRRK2 mutations impair depolarization-induced mitophagy through inhibition of mitochondrial accumulation of RAB10. Autophagy.

[45] Heo J.-M., Ordureau A., Swarup S., Paulo J.A., Shen K., Sabatini D.M., Harper J.W. (2018). RAB7A phosphorylation by TBK1 promotes mitophagy via the PINK-PARKIN pathway. Sci. Adv..

[46] Liang J., Xu Z.-X., Ding Z., Lu Y., Yu Q., Werle K.D., Zhou G., Park Y.-Y., Peng G., Gambello M.J. (2015). Myristoylation confers noncanonical AMPK functions in autophagy selectivity and mitochondrial surveillance. Nat. Commun..

[47] Toyama E.Q., Herzig S., Courchet J., Lewis T.L., Losón O.C., Hellberg K., Young N.P., Chen H., Polleux F., Chan D.C. (2016). AMP-activated protein kinase mediates mitochondrial fission in response to energy stress. Science.

[48] Seabright A.P., Fine N.H.F., Barlow J.P., Lord S.O., Musa I., Gray A., Bryant J.A., Banzhaf M., Lavery G.G., Hardie D.G. (2020). AMPK activation induces mitophagy and promotes mitochondrial fission while activating TBK1 in a PINK1-Parkin independent manner. FASEB J..

[49] Wang B., Nie J., Wu L., Hu Y., Wen Z., Dong L., Zou M.-H., Chen C., Wang D.W. (2018). AMPKα2 Protects Against the Development of Heart Failure by Enhancing Mitophagy via PINK1 Phosphorylation. Circ. Res..

[50] Wang Q., Yang L., Hua Y., Nair S., Xu X., Ren J. (2014). AMP-Activated Protein Kinase Deficiency Rescues Paraquat-Induced Cardiac Contractile Dysfunction Through an Autophagy-Dependent Mechanism. Toxicol. Sci..

[51] Zhou T., Chang L., Luo Y., Zhou Y., Zhang J. (2019). Mst1 inhibition attenuates non-alcoholic fatty liver disease via reversing Parkin-related mitophagy. Redox Biol..

[52] Feng J., Li H., Zhang Y., Wang Q., Zhao S., Meng P., Li J. (2018). Mammalian STE20-Like Kinase 1 Deletion Alleviates Renal Ischaemia-Reperfusion Injury via Modulating Mitophagy and the AMPK-YAP Signalling Pathway. Cell. Physiol. Biochem..

[53] Lv M., Yu B., Mao X.B., Zheng P., He J., Chen D.W. (2012). Responses of growth performance and tryptophan metabolism to oxidative stress induced by diquat in weaned pigs. Animmal.

[54] Mao X.B., Lv M., Yu B., He J., Zheng P., Yu J., Wang Q.Y., Chen D.W. (2014). The effect of dietary tryptophan levels on oxidative stress of liver induced by diquat in weaned piglets. J. Anim. Sci. Biotechnol..

[55] Yin J., Liu M., Ren W., Duan J., Yang G., Zhao Y., Fang R., Chen L., Li T., Yin Y. (2015). Effects of Dietary Supplementation with Glutamate and Aspartate on Diquat-Induced Oxidative Stress in Piglets. PLoS ONE.

[56] Zheng P., Yu B., He J., Yu J., Mao X., Luo Y., Luo J., Huang Z., Tian G., Zeng Q. (2017). Arginine metabolism and its protective effects on intestinal health and functions in weaned piglets under oxidative stress induced by diquat. Br. J. Nutr..

[57] Wang C., Cao S., Shen Z., Hong Q., Feng J., Peng Y., Hu C. (2019). Effects of dietary tributyrin on intestinal mucosa development, mitochondrial function and AMPK-mTOR pathway in weaned pigs. J. Anim. Sci. Biotechnol..

