# Supplementary material for: AMPK-PINK1/Parkin Mediated Mitophagy Is Necessary for Alleviating Oxidative Stress-Induced Intestinal Epithelial Barrier Damage and Mitochondrial Energy Metabolism Dysfunction in IPEC-J2
Source: Antioxidants (Basel). 2021 Dec 18;10(12):2010. doi: 10.3390/antiox10122010 (PMC8698696; doi:10.3390/antiox10122010)
Supplement: Supplementary file 1 [file antioxidants-10-02010-s001.zip › antioxidants-1456617-supplementary.pdf]

Table S1. Primer sequences used for real-time PCR.

Gene	5’-Primer (F)	3’-Primer (R)
β-Actin	CTGCGGCATCCACGAAACT	AGGGCCGTGATCTCCTTCTG
CuZnSOD[1]	CAGGTCCTCACTTCAATCC	CCAAACGACTTCCASCAT
MnSOD[2]	GGACAAATCTGAGCCCTAACG	CCTTGTTGAAACCGAGCC
GPx1[3]	TGGGGAGATCCTGAATTG	GATAAACTTGGGGTCGGT
GPx4[4]	GATTCTGGCCTTCCCTTGC	TCCCCTTGGGCTGGACTTT
BNIP3[5]	GACTCGCCAGGTTACAGACA	CAGTGACGTGCTTAGGACCC
BNIP3L[6]	CCGGAGAACCAGAGACGGTA	GGGCAGAGACTGTTCACCTT
FUNDC-1[7]	GGCAGCACCTGAAATCAACA	GCCTAGCAAAAAGCCTCCCA

[1]*CuZnSOD* = Copper and zinc superoxide dismutase;

[2]*MnSOD* = Manganese-containing superoxide dismutase;

[3]*GPx1* = Glutathione peroxidase 1;

[4]*GPx4* = Glutathione peroxidase 4.

[5]*BNIP3*= BCL2/adenovirus E1B 19 kDa interacting protein 3

[6]*BNIP3L*= BCL2/adenovirus E1B 19 kDa interacting protein 3L

[7]*FUNDC-1*= FUN14 domain containing 1

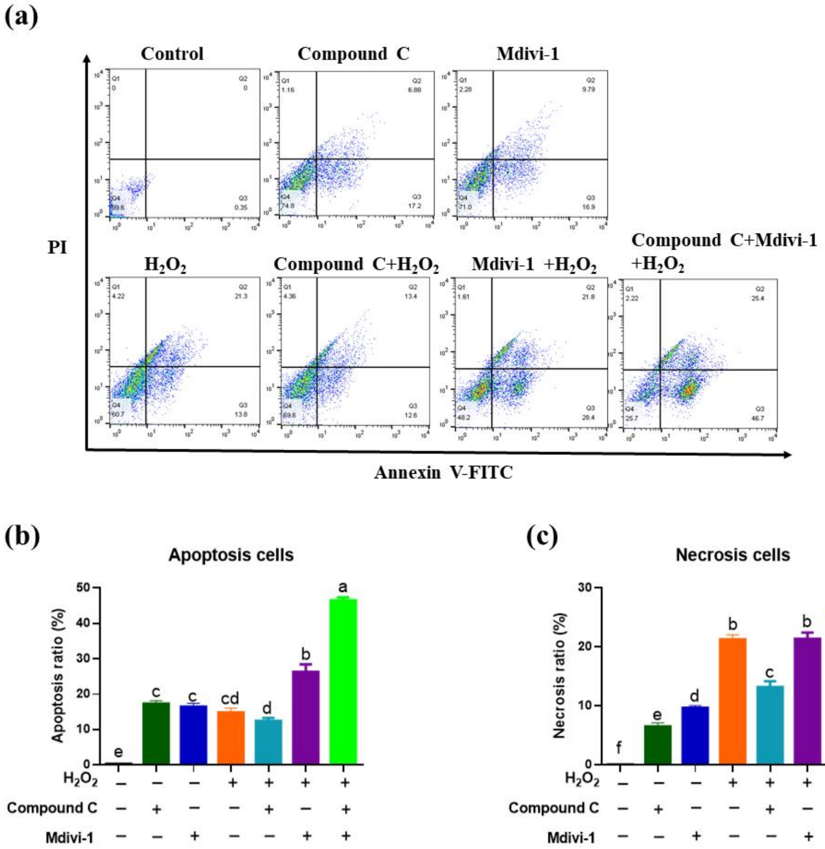

Figure S1. The effect of inhibiting AMPK and mitophagy on the ratio of apoptosis and necrosis cells in H_2O_2-induced oxidative stress model of intestinal epithelial cells. (**a**) In the H_2O_2-induced IPEC-J2 cell oxidative stress model, the detection of apoptosis using Annexin V-FITC/PI staining and flow cytometry; (**b**) In the H_2O_2-induced IPEC-J2 cell oxidative stress model, the effect of inhibiting AMPK signal pathway and mitophagy on the ratio of apoptosis cells (Annexin V-FITC$^+$ PI$^-$); (**c**) In the H_2O_2-induced IPEC-J2 cell oxidative stress model, the effect of inhibiting AMPK signal pathway and mitophagy on the on the ratio of necrosis cells (Annexin V-FITC$^+$ PI$^+$). Data expressed as average ± SD. [a,b,c,d] Means with different letters differ significantly ($p < 0.05$).

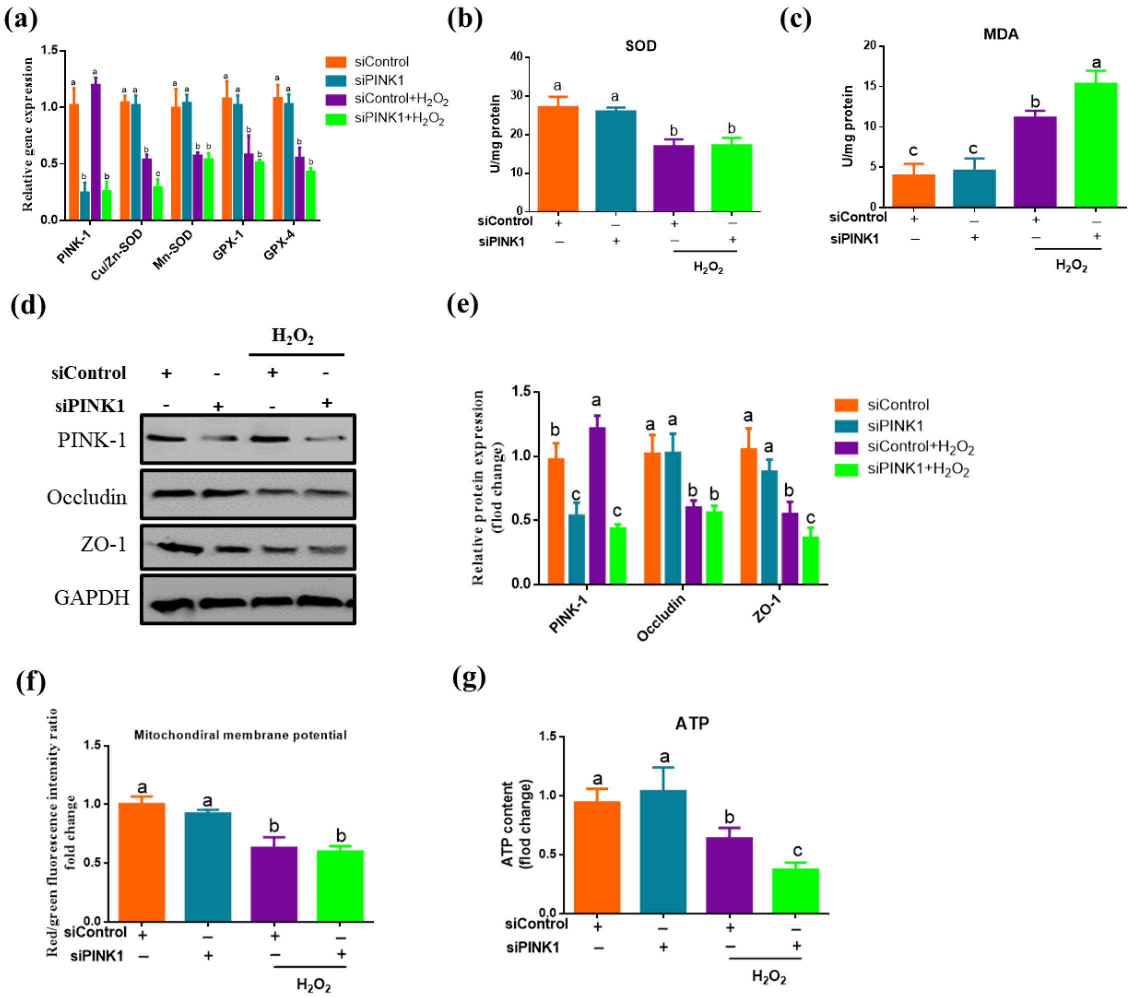

Figure S2. The effects of knockdown of PINK1 in the H_2O_2-induced oxidative stress model of intestinal epithelial cells. (**a**) The expression of *Cu/Zn-SOD, Mn-SOD, GPX-1, GPX-4*; (**b**) SOD activity; (**c**) MDA level; (**d**) Western analysis of PINK1, Occludin and ZO-1 protein expression; (**e**) Relative expression of PINK1, Occludin and ZO-1; (**f**) ATP production; (**g**) mitochondrial membrane potential; Data expressed as average ± SD. [a,b,c] Means with different letters differ significantly ($p < 0.05$).
